# Diarylbibenzofuranone-Based Dynamic Covalent Polymer Gels Prepared via Radical Polymerization and Subsequent Polymer Reaction

**DOI:** 10.3390/gels1010058

**Published:** 2015-07-03

**Authors:** Keiichi Imato, Masamichi Nishihara, Atsushi Irie, Atsushi Takahara, Hideyuki Otsuka

**Affiliations:** 1 Graduate School of Engineering, Kyushu University, 744 Motooka, Nishi-ku, Fukuoka 819-0395, Japan; E-Mails: k-imato@polymer.titech.ac.jp (K.I.); 2 Department of Organic and Polymeric Materials, Tokyo Institute of Technology, 2-12-1 Ookayama, Meguro-ku, Tokyo 152-8550, Japan; 3 Institute for Materials Chemistry and Engineering, Kyushu University, 744 Motooka, Nishi-ku, Fukuoka 819-0395, Japan; E-Mail: nishihara@i2cner.kyushu-u.ac.jp

**Keywords:** self-healing, dynamic covalent chemistry, gels, radical polymerization, polymer reaction

## Abstract

Diarylbibenzofuranone (DABBF) is a dynamic covalent bonding unit, which is in equilibrium with the corresponding radicals at room temperature, and polymers with DABBF linkages show notable properties such as self-healing. The preparation routes have been strictly limited, however, and no polymer with the linkages has been synthesized via radical polymerization because of the strong antioxidant activity of DABBF. Here we present a new method to prepare DABBF-containing polymers via radical polymerization of the precursor, arylbenzofuranone (ABF), and subsequent polymer reaction, dimerization of ABF units in the linear polymers. Polymer gels cross-linked by DABBF linkages were obtained against the relatively strong antioxidant activity of ABF and showed dynamic network reorganization at room temperature.

## 1. Introduction

Self-healing is a very attractive ability in the field of polymers because materials with the intrinsic activity can repair damage done to them without human intervention, which is expected to reduce waste and improve the lifetime, durability, and reliability of the materials. Various approaches to self-healing of polymeric materials have been reported [1], one of which is the utilization of dynamic bonding systems [2]. Healing based on these systems originates from preferential cleavage of dynamic bonds when the material is damaged, subsequent recombination and exchange of the bonds, and resulting reorganization of the polymer networks. Such healing is categorized into two types: healing involving supramolecular chemistry [3], and healing based on dynamic covalent chemistry [4]. Generally, supramolecular systems enable the damaged area to heal under ambient conditions, mostly without external stimuli [5,6,7,8,9,10,11,12]. Alternatively, dynamic covalent systems require appropriate stimuli such as heating, light, and catalyst to induce healing, although the systems behave like usual covalent systems in the absence of the stimuli [13,14,15,16,17,18,19,20,21,22,23,24,25,26,27].

**Figure 1:**
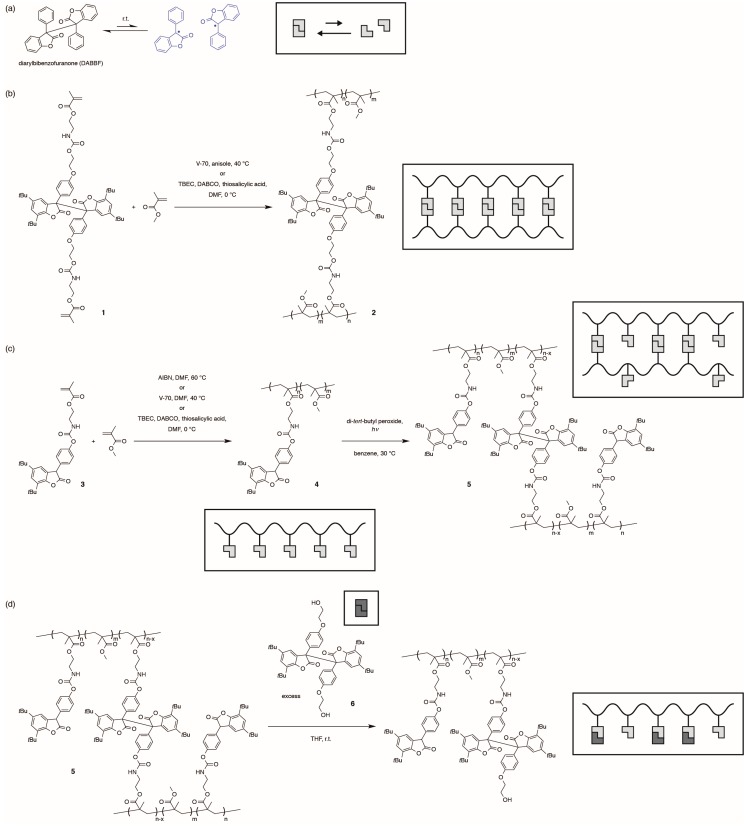
(**a**) Equilibrium between diarylbibenzofuranone (DABBF) and the corresponding arylbenzofuranone (ABF) radicals; (**b**) Synthesis scheme for polymers cross-linked by DABBF linkages via free radical polymerization of DABBF and methyl methacrylate (MMA); (**c**) Synthesis scheme for polymers cross-linked by DABBF linkages via free radical polymerization of ABF-containing methacrylate with MMA and subsequent oxidative coupling reaction; (**d**) De-cross-linking of DABBF-containing cross-linked polymers.

Diarylbibenzofuranone (DABBF) is a unique dynamic covalent-bonding unit because it is in equilibrium in air with the corresponding radicals even at room temperature (Figure 1a) [28,29]. We have found noteworthy properties of DABBF-containing polymers, including self-healing [30,31], temperature-dependent network reorganization [32,33], and damage detection [34,35]. However, polymers incorporating DABBF linkages have been synthesized only via step-growth polymerization (polyaddition) or polymer reaction, and no polymer with the linkages has been prepared via radical polymerization, which is a significant technique in the industry because of its versatility. This is because DABBF shows excellent antioxidant activity ahead of 3,5-di-*tert*-butyl-4-hydroxyanisole, a well-known antioxidant [29,36], and thereby is considered to react with the propagating radical chain ends, terminating the polymerization. Thus, despite the fascinating properties of DABBF-containing polymers, their preparation has been strictly limited to one method.

Here we show a new route to synthesize polymers cross-linked by DABBF linkages via free radical polymerization of a DABBF precursor, arylbenzofuranone (ABF), with a methacryloyl group and subsequent dimerization (cross-linking) of the precursor ABF to form DABBF cross-linkages (Figure 1c). ABF is also known to exhibit antioxidant activity, but it is much lower than that of DABBF [29,37,38]; therefore, radical polymerization is expected to proceed in the presence of ABF and ABF-containing polymers can be available. In this study, we also demonstrated free radical polymerization of dimethacryloyl-functionalized DABBF with methyl methacrylate (MMA) to test the hypothesis (Figure 1b), and we investigated the dynamic properties of gels cross-linked by DABBF linkages for self-healing (Figure 1d).

## 2. Results and Discussion

### 2.1. Radical Copolymerization of DABBF-Containing Bifunctional Monomer and MMA

We prepared dimethacrylate-functionalized DABBF monomer **1** by adding dihydroxy DABBF to two equivalent units of 2-methacryloyloxyethyl isocyanate and allowing them to react. Monomer **1** was employed as a cross-linker for free radical polymerization of MMA, initiated by a low-temperature azo-type initiator, 2,2′-azobis(4-methoxy-2,4-dimethyl valeronitrile) (V-70), in anisole at 40 °C (Figure 1b). As expected, polymerization did not occur at all because of the radical nature of DABBF, which was confirmed by gel-permeation chromatographic (GPC) measurement of the reaction mixture after 45 h. It has been reported that the antioxidant activity of DABBF depends on temperature because increasing temperature biases the equilibrium to the dissociated radicals and, thus, the increased radicals should more easily react with the radical chain ends [36]. Therefore, we also tried to initiate polymerization at a lower temperature, 0 °C, by using a redox-initiation system, where *tert*-butylperoxy 2-ethylhexyl carbonate (TBEC) was employed as an oxidant, and diazabicyclo[2.2.2]octane (DABCO) and thiosalicylic acid were employed as reductants. The reaction at 0 °C for 120 h formed an insoluble component **2** but the yield was quite small, 0.5%, although the concentration was high enough to form a bulk gel (Figure S1). This result indicates that the lower temperature was effective for the radical polymerization in the presence of DABBF, but DABBF was still reactive with the chain ends at 0 °C.

### 2.2. Radical Copolymerization of ABF-Containing Monomer and MMA

**Figure 2:**
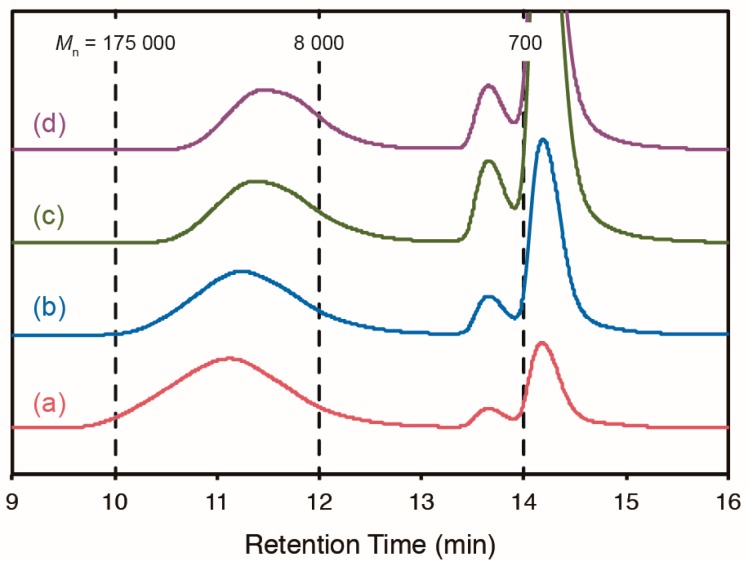
GPC curves of reaction mixtures of entries 5 (**a**), 6 (**b**), 7 (**c**), and 8 (**d**) after polymerization using V-70 as an initiator.

Methacrylate-functionalized ABF monomer **3** was prepared using a method similar to that used for preparing monomer **1**. Monomer **3** and MMA were copolymerized in different ratios at 0, 40, and 60 °C for more than 24 h (Figure 1c). All polymerization was performed in *N*,*N*-dimethylformamide (DMF), a polar aprotic solvent, because the antioxidant activity of ABF (hydrogen abstraction), which is strongly dependent on the solvent polarity, is lower in polar solvents [37], while that of DABBF does not have the same dependence [36]. The hydrogen abstraction from ABF depends on the solvent polarity because it is attributed to the enol form, not the lactone form, and the enol fraction decreases with increasing solvent polarity [37]; meanwhile, the antioxidant activity of DABBF originates from only the dissociated radicals, which have little interaction with solvents [36], and thus its hydrogen abstract does not depend on the solvent polarity. As expected, the polymerization proceeded well at all temperatures. Table 1 summarizes the conditions and results of the polymerization. We obtained ABF-containing polymer **4** with different compositions and with molecular weight of more than 10^4^; in particular, the polymerization at 0 °C produced higher-molecular-weight polymers. However, the compositions of **4** obtained at 0 °C were largely different from the corresponding feed ratios, and insoluble components were observed in the course of the polymerization at 0 °C, indicating that cross-linking or branching, or both cross-linking and branching occurred. The conversion, yield, and molecular weight decreased with increasing ABF ratio in the reaction mixtures; in contrast, the ratio of the component with molecular weight of approximately 10^3^ increased, as shown in Figure 2 and Figure S2. These trends were observed probably because ABF and the generated ABF radicals trapped the propagating radical chain ends and the ABF radicals dimerized to form DABBF, particularly at the beginning of the polymerization.

**Table 1:** Radical copolymerizations of ABF-containing monomer (**3**) and MMA.

Entry	Initiator *^a^*	[3]/[MMA] *^b^*	Temp. (°C)	Time (h)	Conv. (%) *^c^*	Yield (%)	3/MMA *^d^*	*M*_n_^*e*^	*M*_W_/*M*_n_^*e*^
1	AIBN	1:9	60	24	37	29	1:9.0	20,700	1.83
2	AIBN	1:4	60	24	11	5	1:4.1	24,400	1.44
3	AIBN	1:1	60	72	7	7	1:0.5	15,900	1.27
4	AIBN	1:0	60	120	–	3	1:0	14,400	1.19
5	V-70	1:9	40	61	54	40	1:8.8	22,300	1.81
6	V-70	1:4	40	61	33	23	1:3.6	19,000	1.61
7	V-70	1:1	40	61	13	–	–	–	–
8	V-70	1:0	40	120	5	4	1:0	12,300	1.39
9	redox	1:9	0	72	22	14	1:16.0	47,700	2.13
10	redox	1:4	0	72	10	3	1:5.3	34,300	1.75
11	redox	1:1	0	72	12	1	1:0.6	39,700	1.97

*^a^* AIBN = 2,2′-azobisisobutyronitrile; V-70 = 2,2′-azobis(4-methoxy-2,4-dimethylvaleronitrile); redox = *tert*-butylperoxy 2-ethylhexyl carbonate (TBEC), diazabicyclo[2.2.2]octane (DABCO), and thiosalicylic acid. *^b^* Feed ratio; *^c^* Calculated by ^1^H NMR; *^d^* Composition ratio of 4 calculated by ^1^H NMR; *^e^* Determined by GPC in THF.

### 2.3. Cross-Linking of ABF-Containing Linear Polymers by Oxidative Coupling

**Figure 3:**
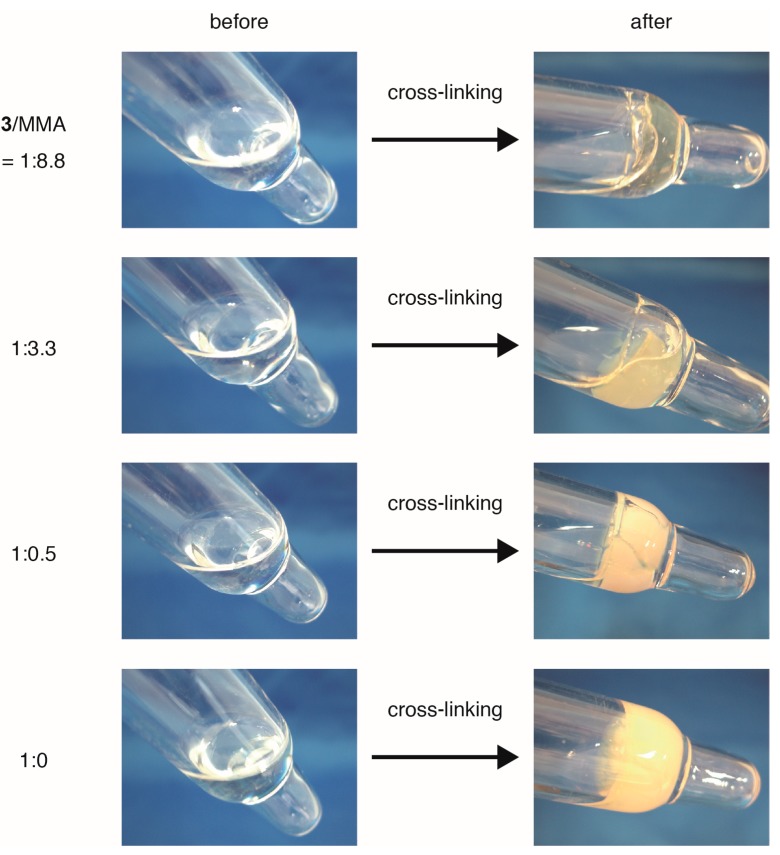
Photographs of linear polymer solutions **4** with different ratios of **3**/MMA (1:8.8, 1:3.3, 1:0.5, and 1:0) before and after cross-linking.

ABF units in the side chains of linear polymers **4** were dimerized to form cross-linked polymers **5** with DABBF linkages (Figure 1c). The linear polymers **4** were irradiated with UV light in the presence of di-*tert*-butyl peroxide in benzene. The radicals generated by UV excitation of di-*tert*-butyl peroxide abstracted hydrogen from the ABF units, and the generated ABF radicals dimerized. Linear polymers **4** formed the corresponding insoluble cross-linked polymers **5** (gels) in any organic solvents in a wide range of composition **3**/MMA (Figure 3). Increases in the glass-transition temperatures (*T*_g_) after the reaction were observed in differential scanning calorimetric (DSC) analysis of the polymers (Table 2), indicating that the DABBF-linked polymers **5** were successfully obtained and cross-linking suppressed the micro-Brownian motion of the polymer chains between the cross-linking points. *T*_g_ of **4** showed a tendency to increase with the ratio of monomer **3**, except for the ABF homopolymer, because the degree of polymerization was quite small (approximately 25). We consider that the intramolecular reaction also occurred in the course of the cross-linking of **4**. However, there is no way to investigate it because of the dynamic nature of the DABBF linkages at room temperature.

**Table 2:** Cross-linking of linear polymer **4**.

3/MMA ^*a*^	*M*_n_^*b*^	*T*_g_ before Cross-Linking (°C) *^c^*	*T*_g_ after Cross-Linking (°C) *^c^*
1:8.8	22,300	108	120
1:3.6	19,000	111	126
1:0.5	15,900	113	131
1:0	12,300	105	130

*^a^* Composition ratio of **4** calculated by ^1^H NMR; *^b^* Determined by GPC in THF; *^c^* Determined by DSC.

### 2.4. De-Cross-Linking of Polymers with DABBF Cross-Linkages

**Figure 4:**
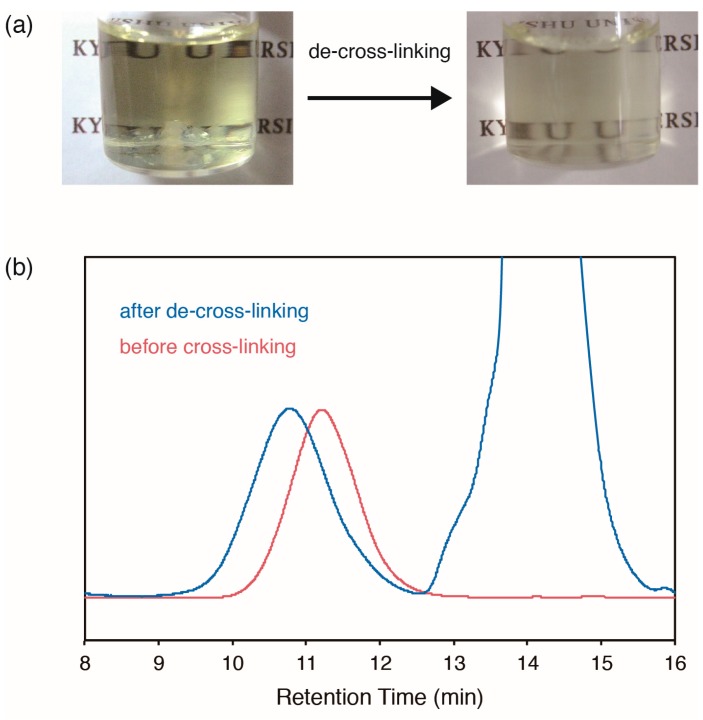
(**a**) Photographs of cross-linked polymers **5** before and after de-cross-linking; (**b**) GPC curves of ABF-containing polymer **4** before cross-linking (red line) and the reaction mixture after de-cross-linking (blue line).

To investigate the dynamic properties of the cross-linked polymers **5**, we performed the de-cross-linking reaction using an excess of low-molecular DABBF **6** (Figure 1d). DABBF units are known to exchange their bonds at room temperature even when they are incorporated in polymer chains, which is the basis of self-healing by dynamic bonding systems [30,31,32,33]. The cross-linked polymers **5** were swollen with a solution of **6** (10 equiv./ABF units) in tetrahydrofuran (THF), forming gels. After waiting for more than four days in air at room temperature, the cross-linked polymers **5** completely dissolved (Figure 4a) and a THF-soluble high-molecular-weight component was detected by GPC measurement of each reaction mixture (Figure 4b). The products were expected to be linear polymers or cross-linked oligomers, or a mixture of both, all with slightly higher molecular weight than the precursor linear polymers **4** because of the cross-links and capping of the side chains by **6**. Therefore, we confirmed that the incorporated DABBF linkages in cross-linked polymers **5** autonomously exchanged their bonds in air at room temperature and the polymer networks reorganized, which would lead to self-healing. Although we tried to perform a detailed analysis by fractionating this component, the cross-linking reaction occurred again after fractionation.

Finally, we estimated the conversion ratio resulting from the polymer reaction (cross-linking) from ^1^H NMR measurements of the reaction mixtures after de-cross-linking (Figure S3). As shown in Table 3, approximately 60% of ABF units in linear polymers **4** dimerized in the course of the reaction, which was not dependent on the composition.

**Table 3:** Degree of cross-linked DABBF in **5**.

3/MMA *^a^*	Cross-linked DABBF (%) *^b^*
1:8.8	53
1:3.6	63
1:0.5	56
1:0	60

*^a^* Composition ratio of **4** calculated by ^1^H NMR; *^b^* Determined by ^1^H NMR of reaction mixtures after de-cross-linking.

## 3. Conclusions

We successfully developed a method to prepare DABBF-containing polymers via radical polymerization and subsequent polymer reaction. The strategy was based on the difference between the reactivity of DABBF and ABF. As anticipated, the strong antioxidant activity of DABBF completely prevented radical polymerization, but that of ABF did not have much of this effect. The polymer reaction—dimerization of ABF units in the linear polymers—generated insoluble gels, in which approximately 60% of the ABF units were cross-linked. Because the DABBF linkages in the gels were in equilibrium and autonomously exchanged their bonds at room temperature, the gels showed the unique dynamic behavior of network reorganization at room temperature, which is necessary for self-healing. The present route for preparation of DABBF-containing polymers using radical polymerization would expand the availability of their intriguing properties, such as self-healing and damage visualization, from daily necessities to engineering materials.

## 4. Experimental Section

### 4.1. Radical Copolymerization of DABBF-Containing Bifunctional Monomer and MMA

In a typical run, a solution of **1** (0.50 g, 0.47 mmol) and MMA (0.94 mL, 8.9 mmol) in DMF, a solution of TBEC (8.7 mg, 35 µmol), a solution of DABCO (0.9 mg, 7.9 µmol) in DMF, and a solution of thiosalicylic acid (8.0 mg, 52 µmol) in DMF after freeze–pump–thaw degassing were combined in a test tube to reach a ratio of DMF/MMA = 1:1 (*v*/*v*). The tube was sealed and the reaction was allowed to proceed at 0 °C for 120 h. After the mixture was reprecipitated from an excess of methanol, filtered, and dried *in vacuo*, DABBF-containing cross-linked polymer **2** was obtained as a white solid (6.5 mg, 0.5% yield).

### 4.2. Radical Copolymerization of ABF-Containing Monomer and MMA

In a typical run, **3** (0.13 g, 0.26 mmol), MMA (0.25 mL, 2.4 mmol), V-70 (2.4 mg, 7.8 µmol), and DMF (0.25 mL) were combined in a test tube. After the freeze–pump–thaw degassing cycle, the tube was sealed and the reaction was allowed to proceed at 40 °C for 72 h. After reprecipitation of the mixture from an excess of methanol, filtered, and dried *in vacuo*, ABF-containing linear polymer **4** was obtained as a white solid (0.14 g, 40% yield). ^1^H NMR (300 MHz, CDCl_3_): δ/ pm 0.70–1.22 (br, α-CH_3_), 1.23–1.50 (br, CH_3_), 1.70–2.10 (br, CH_2_), 3.40–3.80 (br, CH_2_), 4.13 (br, NH), 4.84 (s, CH), 7.07–7.32 (m, aromatic). FT-IR (KBr, cm^−^^1^): 3380, 2990, 2950–2840, 1810–1800, 1740–1720, 1610, 1500–1440, 1270–1240, 1150, 1070, 990, 900, 750.

### 4.3. Cross-Linking of ABF-Containing Linear Polymers

In a typical run, **4** (50 mg, *M*_n_ = 22,300, *M*_W_/*M*_n_ = 1.81, **3**/MMA = 1:8.8), di-*tert*-butyl peroxide (0.10 mL, 0.55 mmol), and benzene (0.51 mL) were combined in a test tube. The solution was irradiated by UV light (high-pressure mercury lamp) for 120 min at 30 °C. After washing with an excess of benzene and freeze-drying, DABBF-containing cross-linked polymer **5** was obtained as a white solid (52 mg, quantitative).

### 4.4. De-Cross-Linking of Gels with DABBF Cross-Linkages

First, **5** (20 mg) and a THF solution (4.5 mL) of **6** (10 equiv./ABF units) were charged into a sample tube. The mixture was then stirred under air at room temperature. After more than four days, the reaction mixture was evaluated by GPC measurements and ^1^H NMR measurements.

## Supplementary Materials

http://www.mdpi.com/2310-2861/1/01/58/s1
