# Supplementary material for: Diarylbibenzofuranone-Based Dynamic Covalent Polymer Gels Prepared via Radical Polymerization and Subsequent Polymer Reaction
Source: Gels. 2015 Jul 3;1(1):58–68. doi: 10.3390/gels1010058 (PMC6318614; doi:10.3390/gels1010058)
Supplement: Supplementary file 1 [file gels-01-00058-s001.pdf]

Supplementary Information

Materials and Methods

Materials

All solvents and reagents used for synthesis and characterization were purchased from Sigma-Aldrich, Wako Pure Chemical Industries, Tokyo Chemical Industry, Kanto Chemical, or Kishida Chemical and used as received, unless otherwise noted. Methyl methacrylate (MMA) and *N*,*N*-dimethylformamide (DMF) were purified by distillation under reduced pressure over calcium hydride. Dimethacrylate-functionalized diarylbibenzofuranone (DABBF) **1** and methacrylate-functionalized arylbenzofuranone (ABF) **3** were synthesized from 4-hydroxymandelic acid and 2,4-di-*tert*-butylphenol in four and two steps, respectively.

Characterization

^{1}H NMR spectroscopic measurements were carried out at 25 °C using a 300 MHz JEOL spectrometer with tetramethylsilane (TMS) as internal standard in chloroform-*d* ($CDCl_3$). IR spectra were obtained with a Perkin-Elmer Spectrum One infrared spectrometer as thin films with KBr. Gel permeation chromatographic (GPC) measurements were carried out at 40 °C on TOSOH HLC-8220 GPC system equipped with a guard column (TOSOH TSK guard column Super H-L), three columns (TOSOH TSK gel SuperH 6000, 4000, and 2500), a differential refractive index detector, and a UV-VIS detector. Tetrahydrofuran (THF) was used as the eluent at a flow rate of 0.6 mL/min. Polystyrene (PS) standards (M_n = 4920–3000000; M_w/M_n = 1.02–1.03) were used to calibrate the GPC system. Differential scanning calorimetric (DSC) measurements were carried out using a SII Nanotechnology DSC6220, with a heating rate of 10 °C/min.

Synthesis of Diarylbibenzofuranone 1

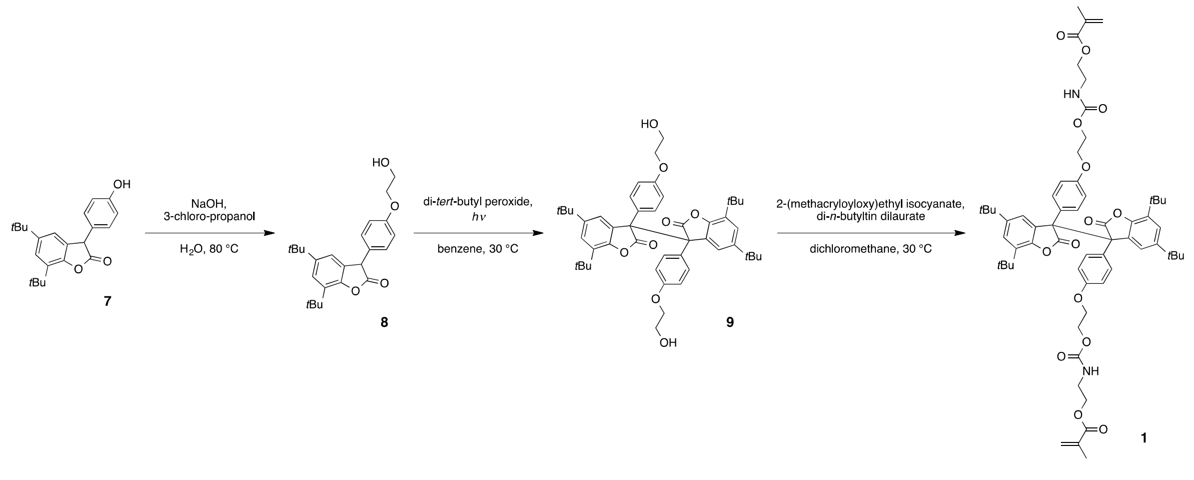

Arylbenzofuranone 7. 7 was synthesized from 4-hydroxymandelic acid and 2,4-di-*tert*-butylphenol according to our previously published method [30].

Arylbenzofuranone 8. In a two neck round bottom flask, a solution of sodium hydroxide (8.12 g, 203 mmol) in water (180 mL) was formed. With stirring, **7** (20.2 g, 59.6 mmol) was added and the mixture heated to 80 °C under a nitrogen atmosphere. Once at 80 °C, 2-chloroethanol (7.00 mL,

104 mmol) was added and the reaction held at 80 °C for three hours. After cooling to room temperature, a solution of concentrated hydrochloric acid (21.2 mL, 240 mmol) in water (249 mL) was added and the reaction held again at 80 °C for an additional hour. Once cooled to room temperature, the liquid was decanted and the remaining solid dissolved in dichloromethane (DCM). This solution was washed several times with water and saline solution. After drying the DCM layer over anhydrous magnesium sulfate and concentrating, the crude product was purified by flash chromatography eluting with ethyl acetate/hexane (2/3, *v*/*v*) and dried *in vacuo* to give ABF **8** as a white solid (14.7 g, 65% yield). ^{1}H NMR (300 MHz, $CDCl_3$): δ/ ppm 1.29 (s, 9H, CH_3), 1.43 (s, 9H, CH_3), 3.95–3.99 (m, 2H, CH_2), 4.07–4.09 (m, 2H, CH_2), 4.79 (s, 1H, CH), 6.92 (d, J = 9 Hz, 2H, aromatic), 7.04 (s, 1H, aromatic), 7.17 (d, J = 9 Hz, 2H, aromatic), 7.23–7.32 (m, 1H, aromatic). FT-IR (KBr, cm^{-1}): 3570–3430, 3060–3040, 2960–2870, 1810–1790, 1610–1590, 1510, 1480, 1250, 1080, 910, 820.

Diarylbibenzofuranone 9. **8** (500 mg, 1.31 mmol), di-*tert*-butyl peroxide (2.76 mL, 15.0 mmol) and benzene (4.10 mL) were combined in a test tube. The solution was irradiated with UV light for 60 min at 30 °C. After concentration of the solution, the crude product was purified by flash chromatography eluting with ethyl acetate/hexane (3/2, *v*/*v*) and dried *in vacuo* to give DABBF **9** as a yellow solid (283 mg, 57% yield). ^{1}H NMR (300 MHz, $CDCl_3$): δ/ ppm 1.03–1.43 (m, 36H, CH_3), 3.96–4.16 (m, 8H, CH_2), 6.35 (br, 2H, OH), 6.77–7.30 (m, 12H, aromatic). FT-IR (KBr, cm^{-1}): 3430, 3080–3040, 2960–2870, 1800–1790, 1610, 1510, 1260, 1090, 920, 820.

Diarylbibenzofuranone 1. In a round bottom flask, **9** (1.98 g, 2.59 mmol) was added with stirring and dried *in vacuo*. DCM (12 mL), di-*n*-butyltin dilaurate (20.0 μL, 30.0 μmol), and 2-(methacryloyloxy)ethyl isocyanate (0.80 mL, 5.66 mmol) were dropped into the mixture via syringe under a nitrogen atmosphere at 30 °C and the reaction held at 30 °C for 34 h. After that, the solution was concentrated and the crude product was purified by flash chromatography eluting with ethyl acetate/hexane (3/1, *v*/*v*). After recrystallizing from chloroform and hexane, DABBF **1** was obtained as a yellow solid (2.64 g, 95% yield). ^{1}H NMR (300 MHz, $CDCl_3$): δ/ ppm 1.03–1.50 (m, 36H, CH_3), 1.93 (s, 6H, CH_3), 3.52 (d, J = 8 Hz, 4H, CH_2), 4.18–4.25 (m, 8H, CH_2), 4.44 (s, 4H, CH_2), 5.07 (br, 2H, NH), 5.58 (s, 2H, vinyl proton), 6.11 (s, 2H, vinyl proton), 6.81–7.29 (m, 12H, aromatic). FT-IR (KBr, cm^{-1}): 3590–3380, 3080, 2960–2870, 1800, 1740–1700, 1640–1610, 1510, 1250, 1180, 1080, 900.

Synthesis of Arylbenzofuranone 3

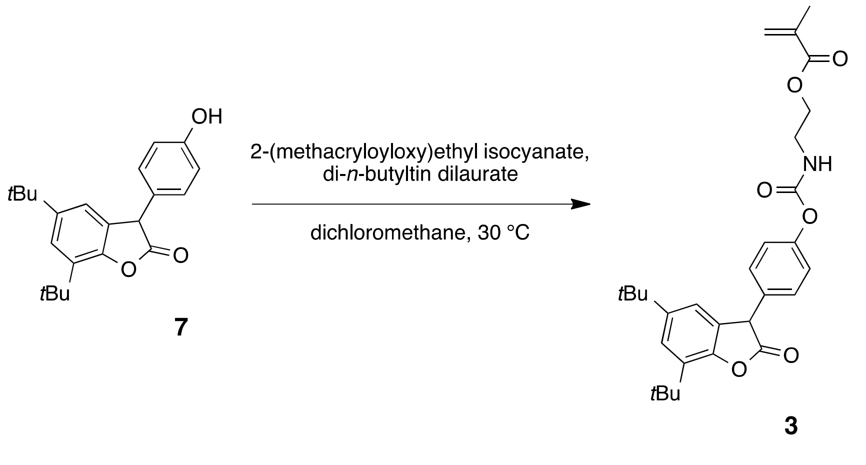

Arylbibenzofuranone 3. In a round bottom flask, **7** (10.5 g, 31.0 mmol) was added with stirring and dried *in vacuo*. DCM (60 mL), di-*n*-butyltin dilaurate (20.0 µL, 30.0 µmol), and 2-(methacryloyloxy) ethyl isocyanate (8.80 mL, 62.2 mmol) were dropped into the mixture via syringe under a nitrogen atmosphere at 30 °C and the reaction held at 30 °C for 24 h. After that, the solution was concentrated and the crude product was purified by flash chromatography eluting with ethyl acetate/hexane (2/1, *v*/*v*). After recrystallizing from chloroform and hexane, ABF **3** was obtained as a white solid (14.0 g, 91% yield). ^{1}H NMR (300 MHz, $CDCl_3$): δ/ppm 1.30 (s, 9H, CH_3), 1.43 (s, 9H, CH_3), 1.97 (s, 3H, CH_3), 3.60 (td, J = 6 Hz, 2H, CH_2), 4.31 (t, J = 5 Hz, 2H, CH_2), 4.83 (s, 1H, CH), 5.32–5.33 (br, 1H, NH), 5.63 (s, 1H, vinyl proton), 6.16 (s, 1H, vinyl proton), 7.07 (s, 1H, aromatic), 7.14 (d, J = 9 Hz, 2H, aromatic), 7.25 (d, J = 9 Hz, 2H, aromatic), 7.33 (s, 1H, aromatic). FT-IR (KBr, cm^{-1}): 3590–3350, 3040, 2960–2870, 1810–1800, 1740–1720, 1640, 1500, 1220, 1170, 1080, 900.

Radical Polymerization of Diarylbibenzofuranone

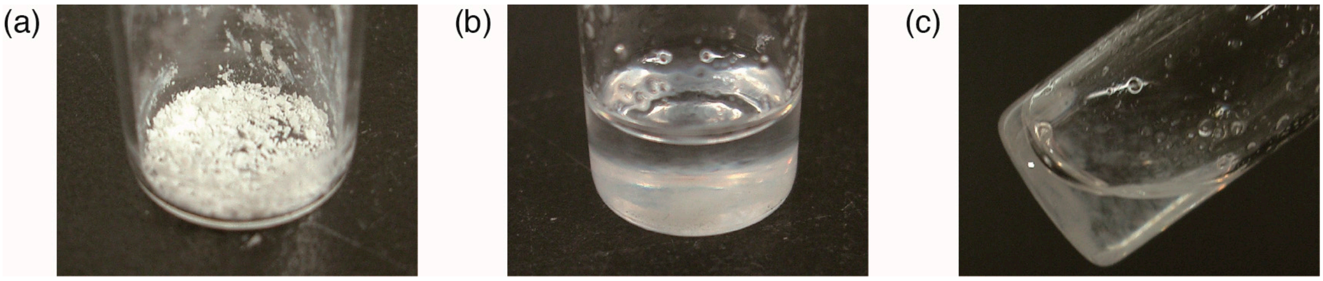

Figure S1. Photographs of cross-linked polymer **2** after purified (**a**) and swollen with anisole (**b**, **c**).

Radical Polymerization of Arylbenzofuranone

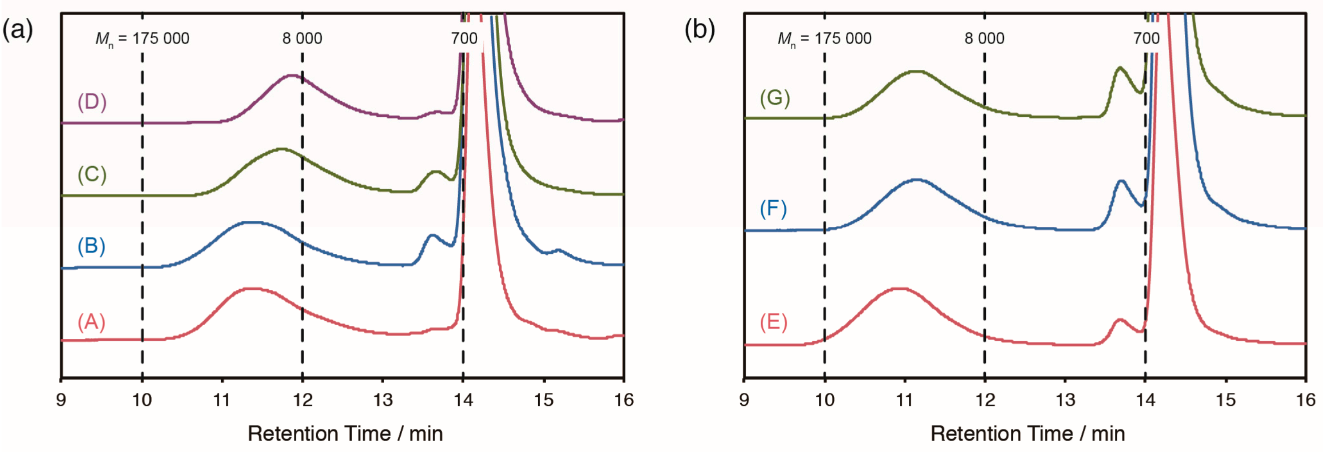

Figure S2. GPC curves of reaction mixtures after polymerization using AIBN (**a**) and a redox system (**b**) as an initiator: entries 1 (A), 2 (B), 3 (C), 4 (D), 9 (E), 10 (F), and 11 (G).

De-Cross-Linking of DABBF-Containing Polymers

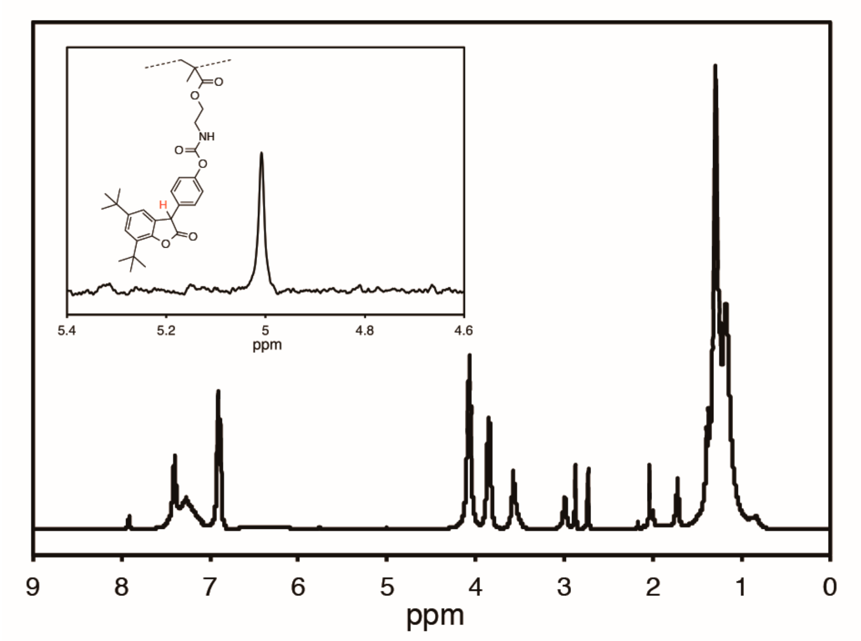

Figure S3. ^{1}H NMR spectrum of a reaction mixture after de-cross-linking of **5**. The insert shows a spectrum magnified. The conversions of cross-linking were calculated from the peak at **5** ppm, which originates from unreacted ABF units in **5**.
